# Frequency-Temporal Disagreement Adaptation for Robotic Terrain Classification via Vibration in a Dynamic Environment

**DOI:** 10.3390/s20226550

**Published:** 2020-11-16

**Authors:** Chen Cheng, Ji Chang, Wenjun Lv, Yuping Wu, Kun Li, Zerui Li, Chenhui Yuan, Saifei Ma

**Affiliations:** 1 Department of Automation, University of Science and Technology of China, Hefei 230027, China; 2014107@ahiib.edu.cn (C.C.); cjchange@mail.ustc.edu.cn (J.C.); zkdlk@mail.ustc.edu.cn (K.L.); lzerui@mail.ustc.edu.cn (Z.L.); 2 School of Information Engineering, Anhui Institute of International Business, Hefei 231131, China; 3 Key Laboratory of Industrial Computer Control Engineering of Hebei Province, Yanshan University, Qinhuangdao 066004, China; ypwu@stumail.ysu.edu.cn; 4 Department of Research and Development, Anhui Etown Information Technology Co., Ltd, Hefei 230011, China; 5 Institute of Artificial Intelligence, Hefei Comprehensive National Science Center, Hefei 230088, China; yuanch@stu.ahu.edu.cn (C.Y.); sfma@stu.ahu.edu.cn (S.M.); 6 School of Computer Science and Technology, Anhui University, Hefei 230601, China

**Keywords:** autonomous robot, non-geometric hazards, terrain classification, dynamic environment, vibration

## Abstract

The accurate terrain classification in real time is of great importance to an autonomous robot working in field, because the robot could avoid non-geometric hazards, adjust control scheme, or improve localization accuracy, with the aid of terrain classification. In this paper, we investigate the vibration-based terrain classification (VTC) in a dynamic environment, and propose a novel learning framework, named DyVTC, which tackles online-collected unlabeled data with concept drift. In the DyVTC framework, the exterior disagreement (ex-disagreement) and interior disagreement (in-disagreement) are proposed novely based on the feature diversity and intrinsic temporal correlation, respectively. Such a disagreement mechanism is utilized to design a pseudo-labeling algorithm, which shows its compelling advantages in extracting key samples and labeling; and consequently, the classification accuracy could be retrieved by incremental learning in a changing environment. Since two sets of features are extracted from frequency and time domain to generate disagreements, we also name the proposed method feature-temporal disagreement adaptation (FTDA). The real-world experiment shows that the proposed DyVTC could reach an accuracy of 89.5%, but the traditional time- and frequency-domain terrain classification methods could only reach 48.8% and 71.5%, respectively, in a dynamic environment.

## 1. Introduction

Robotic terrain classification refers to the process of a mobile robot classifying the terrain, on which it is traversing or will traverse, as one of the predefined classes [1]. An accurate terrain classification method is of great importance to an autonomous robot performing field tasks which usually need to traverse a variety of terrains like sand, grass, gravel, or clay [2,3]. For example, if  a wheeled robot decides to traverse the sandy ground, its wheels may sink into the sand; and therefore, the robot could only move at an extreme low speed or even be trapped. To prevent the robots from suffering from such non-geometric hazards, the mobile robots must have the ability of terrain classification [4]. For another example, the robotic pose estimation, calculated by the kinematics model, which includes the slip parameters decided by the traversing terrains, usually benefits a lot from robotic terrain classification, especially in the situation without reliable global positioning systems [5,6,7,8]. Apart from hazards avoidance and pose estimation, many existing works have demonstrated that the performances of many other robotic fundamental functions, such as energy savings, route planning, gait control, etc., can be improved significantly from an accurate terrain classification method [9,10,11,12,13]. Therefore, terrain classification and its relevant research have received great attention from the DARPA Grand Challenge and Mars Exploration Plan [14].

As a non-interactive approach, the visual terrain classification method can recognize not only the traversing terrain, but also the terrains traversed or that will be traversed [15,16,17]. However, it suffers from two issues: (i) vision cannot work in extreme illumination (glare or dark); (ii) vision may be confused by the covering materials, thus it cannot recognize the real terrain [18,19,20]. Therefore, the interactive terrain classification, which are often implemented by means of acoustics [21,22], haptics [23,24], or vibration [25,26], is becoming more and more promising in robotic environment perception. The acoustic terrain classification has not been intensively studied, because its robustness against environmental noises cannot be guaranteed [27]. The haptic terrain classification is usually realized by means of tactile sensor arrays mounted on the robot–terrain contact area, thus it is more suitable for legged robots [28]. More than sound and contact force, the robot–terrain interaction generated vibration provides sufficient information to discriminate different types of terrains [29]. The time series collected by an accelerometer is the mixture of vibration and gravitational acceleration. As a result that gravity is almost time-invariant, the vibration can be easily recovered from the accelerometer readings; and therefore, the vibration-based terrain classification (VTC) method has incomparable advantages over the acoustic one. Additionally, unlike the haptic one, the VTC can be applied to both wheeled and legged robots. Hence, this paper concentrates on the vibration-based terrain classification.

Although a large body of terrain classification methods based on VTC have been investigated, most of them are achieved by supervised learning without considering the unlabeled upcoming vibration data [25,30,31,32,33,34]. In fact, we cannot guarantee a sufficient sampling of training dataset, so it is nature to resort to the semi-supervised or unsupervised machine learning tools for VTC. This idea was first proposed for safely operation of planetary exploration rovers [35]. In their work, co- and self-training approaches are employed, and two modalities, vibration and vision, are used to constitute two independent views, thus enabling the vibration- and vision-based classifiers to learn and develop mutually. Meanwhile, the vision-based classifier learns by itself when visions are collected on the different patches of vibration. More work that concerns the semi-supervised or unsupervised learning applying to the field of terrain classification can be found in [36,37,38]. These methods could be used in static environments, where the offline training dataset and the online testing dataset are independent and identically distributed (iid). However, if the training dataset is obtained from a certain area of grassland and the testing dataset from another, it is highly possible that the two datasets are non-iid since the two areas differ in moisture, roughness, or some other aspects. Hence, the dynamic environment could give rise to a degradation in predicting incoming vibration samples by using the classifier learned from the offline training dataset [39].

In this paper, we propose a vibration-based terrain classification framework for autonomous robots working in a dynamic environment (named DyVTC), mainly to suppress the affect rendered by data drift, during the period that manual labels do not arrive. First, according to different feature extraction methods, we construct the time- and feature-domain classifiers from the vibration view. Second, considering the potential temporal correlation in the traversed terrain patches, we introduce the Bayesian filter to correct the terrain predictions output by the two classifiers. Third, in terms of the classifier- and filter-output terrain predictions of the two domains, we propose a novel disagreement-based learning algorithm, which can be read as the most valuable contribution of our work. In the learning algorithm, the concept of ex- and in-disagreement are introduced, which is verified to be powerful to extract key samples and label them in high accuracy.

The rest of the paper is organized as follows. Section 2 covers the framework description of the proposed terrain classification method, as well as the details of some key steps, including feature extraction, classification algorithm, Bayesian filter, domain fusion, and pseudo-labeling algorithm. Section 3 presents the experimental verification, including the description of the experimental robot and data collection, performance evaluation of classifier and Bayesian filter, and comparative study between the existing methods and ours. The paper is concluded in Section 4.

## 2. Methodology

**Figure 1:**
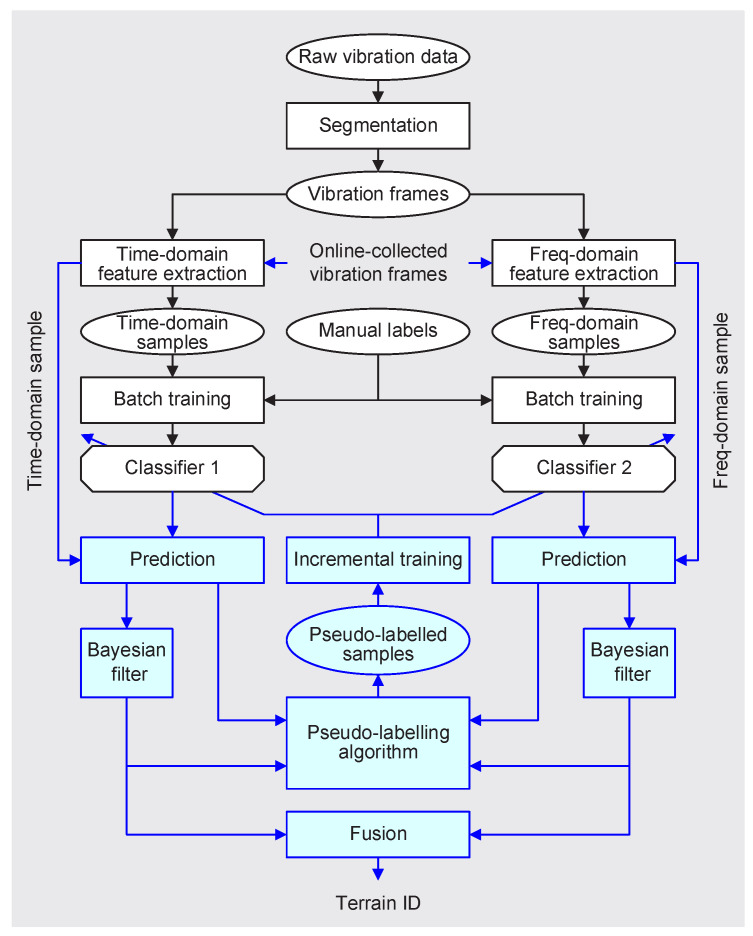
Framework of the proposed dynamic vibration-based terrain classification (DyVTC). The rectangular and elliptical blocks represent *operations* and *dataset*, respectively. The rectangles without corners represent *models*. We use the color blue to highlight the online parts.

The framework of the proposed DyVTC is shown in Figure 1. A single vibration point provides an extreme limited information, so we should use a vibration frame, which is composed of a certain number of successive vibration points, to extract its representative features. All vibration frames are transformed into samples both in the time and frequency domain. Based on the labeled time- and frequency-domain vibration samples, two classifiers are obtained by batch training, respectively. The above process is offline. When the mobile robot is operating outdoors, online-collected vibration samples are fed into the pre-trained classifiers; and then, the classifier-output terrain predictions are fed into Bayesian filter to yield a better terrain prediction. Meanwhile, the  classifier- and filter-output terrain predictions are analyzed based on the mechanism of ex- and in-disagreement; and therefore, some key samples could be extracted and labeled in high accuracy. When these pseudo-labeled samples accumulate to some extent, they are used to re-train the classifiers incrementally. The rest of the section expatiates on some key steps in the DyVTC.

### 2.1. Feature Extraction

We use an accelerometer to detect the acceleration along the vertical axis at 100 Hz, thus obtaining the acceleration time series. Due to the presence of gravity, the accelerometer does not detect a pure motion vibration, but  the vertical acceleration mixed with gravitational acceleration. Hence, we subtract the gravitational acceleration constant from the acceleration time series, and therefore obtain the vibration time series. Furthermore, the vibration time series is split into vibration frames, each of which contains *n* vibration points. To guarantee a real-time terrain classification, each vibration frame overlaps the successive one by 50%. Define a vibration frame by $a=(a_{1},a_{2},\cdots ,a_{n})$. Now we are in the position to extract features from *a* in the frequency domain and time domain.

#### 2.1.1. Frequency-Domain Features

The expression of time series in the frequency domain is usually beneficial to simplify the mathematical analysis and understand the signal components. The discrete Fourier transform (DFT) is such a powerful tool to yield the amplitude spectrum of the time series, thus being intensively used in the analysis of time series. The *N*-point DFT on the vibration frame *a* is defined by [40]

(1) $$A_{k}=\sum_{i=0}^{N-1}a_{i}e^{-j\frac{2\pi ki}{N}},k=0,1,...,N-1,$$

where $j^{2}=-1$, *k* is the frequency. The implementation of DFT often employs an efficient algorithm, which is well known as fast Fourier transform (FFT). For an *N*-point FFT, the parameter *N* is typically specified as a power of 2 or a value that can be factored into a product of small prime numbers. In the case N>n, the vibration frame *a* should be padded using zeros; that is, the terms from $a_{n+1}$ to $a_{N}$ are specified as zeros.

The accelerometer usually work at a frequency of up to 100 Hz. If the terrain classification is desired to work at 1 Hz, which means the prediction should be given every second, then we use the 128-point FFT to transform the vibration frames into their spectrums. If treating the spectrum as the feature directly, the feature is a 128-dimensional vector. In order to reduce the feature dimension, we sample some entries uniformly from the spectral vector to constitute the feature.

#### 2.1.2. Time-Domain Features

Other than the frequency domain, we also extract the features in the time domain directly. A 10-dimensional feature vector $\phi =(\phi_{1},\phi_{2},\cdots ,\phi_{10})$ is obtained, and its entries are shown in Table 1. It is noted that $\phi_{5}$ can be extended by setting τ=1,2,⋯,n−1. However, according to the Khintchine’s law, it should be guaranteed that τ≪n to bound the estimation error of $\phi_{5}$. In this paper, we choose τ=1.

### 2.2. Support Vector Machine

Let $\left\{\left(x_{1},y_{1}\right),...,\left(x_{m},y_{m}\right)\right\}$ denote the training set, where *m* is the size of the training set and $y_{i}\in \left\{\pm 1\right\}$. Support vector machine (SVM) aims to construct a separating hyperplane between two classes of points that maximizes the margin between the hyperplane and support vectors [41]. Usually the hyperplane cannot be found in the original sample space. For such a nonlinear classification task, kernel technique is applied to map the original data to a high-dimensional feature space by φ:x→φ(x). Inner product of points in feature space is then conducted implicitly by a kernel function. In our work, we use two common kernel functions that are linear kernel $\kappa \left(x_{i},x_{j}\right)=x_{i}^{\prime }x_{j}$, and Gaussian kernel $\kappa \left(x_{i},x_{j}\right)=\exp\left(-\frac{∥x_{i}-x_{j}{∥}^{2}}{2\sigma^{2}}\right)$, where σ denotes the width of the Gaussian kernel. Soft margin is used to regularize the trade-off between minimizing the training error and maximizing the margin. Therefore, an SVM can be described as the following optimization problem [42]

(2) $$\begin{array}{cc} \; & \min_{\omega ,b,\xi }\;\frac{1}{2}{∥\omega ∥}^{2}+\lambda \sum_{i=1}^{m}\xi_{i} \end{array}$$

(3) $$\begin{array}{cc} \; & s.t.\;\;y_{i}\left(\omega^{\prime }\varphi \left(x_{i}\right)+b\right)\geq 1-\xi_{i} \end{array}$$

(4) $$\begin{array}{cc} \; & \;\;\;\;\xi_{i}\geq 0,\;i=1,2,...,m \end{array}$$

where ω is the vector normal to the hyperplane, *b* is a scalar bias, and λ is the soft margin parameter. Multi-class classification task of SVM can be performed using one-versus-one approach. The SVM model can be updated online using incremental SVM (i.e., [43]). As a result that only the support vectors participate in the learning process, the incremental SVM reduces the training time greatly and seldom loses accuracy.

### 2.3. Bayesian Filter

The recursive form of Bayesian filter can be seen in [44]. Define $\chi_{t}$ as the state at time *t*, $c_{t}$ the measurement, and $C_{t}=\left\{c_{1},c_{2}\cdots ,c_{t}\right\}$ the measurement set. The purpose is to acquire $P(\chi_{t}|C_{t})$, the *a posteriori* possibility distribution function (pdf) of $\chi_{t}$ conditioned on $C_{t}$. Given $P(\chi_{t-1}|C_{t-1})$, we have    

(5) $$\begin{array}{cc} \; & P\left(\chi_{t}|C_{t-1}\right)=\int P\left(\chi_{t}|\chi_{t-1}\right)P\left(\chi_{t-1}|C_{t-1}\right)d\chi_{t-1}, \end{array}$$

(6) $$\begin{array}{cc} \; & P\left(\chi_{t}|C_{t}\right)=\frac{P\left(c_{t}|\chi_{t}\right)P\left(\chi_{t}|C_{t-1}\right)}{\int P\left(c_{t}|\chi_{t}\right)P\left(\chi_{t}|C_{t-1}\right)d\chi_{t}}, \end{array}$$

where $P(\chi_{t}|C_{t-1})$ denotes the *a priori* pdf of $\chi_{t}$ conditioned on $C_{t-1}$.

Define $\chi_{t}$ as the state at time *t*, $c_{t}$ the measurement, and $C_{t}=\left\{c_{1},c_{2}\cdots ,c_{t}\right\}$ the measurement set. The purpose is to acquire $P(\chi_{t}|C_{t})$, the *a posteriori* possibility distribution function (pdf) of $\chi_{t}$ conditioned on $C_{t}$. Generally speaking, analytic solutions to Equations (5) and (6) are unavailable in most cases, so the estimation problem for continuous state is seldom tackled by Bayesian filter. However, if the state is discrete and its number is not too large, the Bayesian filter is a practicable method to solve such a state estimation problem. In terrain classification, the state at time *t* is defined as $\chi_{t}\in \left\{1,2,\cdots ,\ell \right\}$ where i=1,2,⋯,ℓ denotes the terrain ID. The measurement $c_{t}\in \left\{1,2,\cdots ,\ell \right\}$ is the classifier-output terrain predictions. Given $P(\chi_{t-1}|C_{t-1})$, we have

(7) $$\begin{array}{cc} \; & P\left(\chi_{t}=i|C_{t-1}\right)=\sum_{j=1}^{\ell }P\left(\chi_{t}=i|\chi_{t-1}=j\right)P\left(\chi_{t-1}=j|C_{t-1}\right), \end{array}$$

(8) $$\begin{array}{cc} \; & P\left(\chi_{t}=i|C_{t}\right)=\frac{P\left(c_{t}=j|\chi_{t}=i\right)P\left(\chi_{t}=i|C_{t-1}\right)}{\sum_{i=1}^{\ell }P\left(c_{t}=j|\chi_{t}=i\right)P\left(\chi_{t}=i|C_{t-1}\right)}, \end{array}$$

where $P(\chi_{t}|C_{t-1})$ denotes the *a priori* pdf of $\chi_{t}$ conditioned on $C_{t-1}$, $P(\chi_{t}=i|\chi_{t-1}=j)$ denotes the probability that the mobile robot moves from terrain *j* to *i* at time *t*, and $P(c_{t}=j|\chi_{t}=i)$ denotes the probability of the classifier outputting terrain *j* conditioned on terrain *i*. Meanwhile, we observe that the denominator of Equation (8) is a normalizer.

Applying the Bayesian filter to improve the terrain classification is on the premise of knowing $P(\chi_{0}|C_{0})$, $P(c_{t}|\chi_{t})$ and $P(\chi_{t}|\chi_{t-1})$. First, the initial *a posteriori* pdf $P(\chi_{0}|C_{0})$, where $C_{0}$ denotes a set of no measurements, describes the distribution of the terrain at which the mobile robot locates initially. If the initial terrain is known, then we have $P(\chi_{0}=i|C_{0})=1$ and $P(\chi_{0}\neq i|C_{0})=0$ when locating at terrain *i*; otherwise, $P(\chi_{0}|C_{0})$ is assumed to be uniform distribution, namely, $P\left(\chi_{0}=i|C_{0}\right)=\frac{1}{\ell }$ for i=1,2,⋯,ℓ. Second, $P(c_{t}|\chi_{t})$, which is required during the measurement-update procedure, is determined by the confusion matrix. Third, $P(\chi_{t}|\chi_{t-1})$, which is required during the time-update procedure, describes the correlation of the sampled terrain series. Given *ℓ* terrains, an ℓ×ℓ square matrix *M* with elements $m_{ij}=P\left(\chi_{t}=i|\chi_{t-1}=j\right)$ is defined. The diagonal elements $m_{ii}$ where i=1,2,⋯,ℓ should be assigned a relatively large value not greater than 1, based on the heuristic that terrain is spatially continuous. The off-diagonal elements $m_{ij}$ where i≠j can be determined by the terrain distribution in a map. For example, if terrain *i* possesses more area than terrain *j*, then $m_{ij}<m_{ji}$. It should be guaranteed that the sum of a row equals 1. A general and simple setup of *M* is that $m_{ii}=\mu$ for i=1,2,⋯,ℓ and $m_{ij}=\frac{1-\mu }{\ell -1}$ for i≠j.

### 2.4. Pseudo-Labeling Algorithm

The pseudo-labeling algorithm aims to extract key samples, and label them in a high accuracy. The term *key samples* is denoted as the unlabeled samples that cannot be correctly classified. Now we introduce a new term named *interior disagreement* (*in-disagreement*). For each domain, we have two terrain predictions at the same time. The classifier outputs are read as the *a priori* terrain predictions, while the filter outputs as the *a posteriori* terrain predictions. If the *a priori* and *a posteriori* terrain predictions of the same domain at a certain time are different, then this phenomenon is referred to as *in-disagreement*. The term *a priori ex-disagreement* means the *a priori* terrain predictions at a certain time of the two domains are different. Similarly to the *a priori ex-disagreement*, the *a posteriori ex-disagreement* is denoted by that the *a posteriori* terrain predictions at a certain time of the two domains are different. Based on the in- and ex-disagreement, we propose the following heuristic rules:

1. If one domain (denoted as the 1st domain) appears in-disagreement at a certain time, the sample is likely to be a key sample of the 1st domain.
2. Based on the first rule, if at the same time, the other domain (denoted as the 2nd domain) does not appear in-disagreement, and there is no *a posteriori* ex-disagreement between the two domains, then the 2nd-domain terrain prediction is likely to be a reliable label to the 1st-domain key sample.
3. If in-disagreement appears in both domains, but there is no *a posteriori* ex-disagreement, the filter-output terrain prediction can be used to label the samples from both domains.
4. If neither in-disagreement nor ex-disagreement appears at a certain time, the sample is likely to be classified correctly, thus not a key sample.

Now we present the algorithm in detail. Define γ∈{T,F} as the domain type, where *T* stands for time domain, and *F* for frequency domain. In the γ domain, upon feeding a sample $x_{t}^{\gamma }$, the γ-domain classifier outputs the *a priori* terrain prediction $c_{t}^{\gamma }$; and then, the Bayesian filter outputs the *a posteriori* terrain prediction ${\hat{c}}_{t}^{\gamma }$. The pseudo-labeling algorithm is shown in Algorithm 1. As a result that the rules are proposed on the mechanism of in- and ex-disagreement, we name it in- and ex-disagreement-based pseudo-labeling (IE). The proposed IE is a sample that is an efficient method to extract and label key samples, which will be verified in Section 3.
**Algorithm 1** In- and Ex-Disagreement-Based Pseudo-Labeling Algorithm (IE)**Input:** The unlabeled samples $x_{t}^{T}$ and $x_{t}^{F}$, the *a priori* terrain predictions $c_{t}^{T}$ and $c_{t}^{F}$, the *a posteriori* terrain predictions ${\hat{c}}_{t}^{T}$ and ${\hat{c}}_{t}^{F}$, where t=1,2,⋯,K.
**Output:** Pseudo-labeled sample sets $L_{T}$ and $L_{F}$, for time and frequency domain, respectively.
1:set $L_{T},L_{F}\leftarrow Ø$2:**for**t=1 to *K* **do**3:   **if** $c_{t}^{T}={\hat{c}}_{t}^{T}$ and $c_{t}^{F}\neq {\hat{c}}_{t}^{F}$ and ${\hat{c}}_{t}^{T}={\hat{c}}_{t}^{F}$ **then**4:     
$L_{F}\leftarrow L_{F}\cup \left(x_{t}^{F},{\hat{c}}_{t}^{T}\right)$
5:  
**end if**
6:   **if** $c_{t}^{F}={\hat{c}}_{t}^{F}$ and $c_{t}^{T}\neq {\hat{c}}_{t}^{T}$ and ${\hat{c}}_{t}^{T}={\hat{c}}_{t}^{F}$ **then**7:     
$L_{T}\leftarrow L_{T}\cup \left(x_{t}^{T},{\hat{c}}_{t}^{F}\right)$
8:  
**end if**
9:   **if** $c_{t}^{T}\neq {\hat{c}}_{t}^{T}$ and $c_{t}^{F}\neq {\hat{c}}_{t}^{F}$ and ${\hat{c}}_{t}^{T}={\hat{c}}_{t}^{F}$ **then**10:     $L_{F}\leftarrow L_{F}\cup \left(x_{t}^{F},{\hat{c}}_{t}^{T}\right)$, $L_{T}\leftarrow L_{T}\cup \left(x_{t}^{T},{\hat{c}}_{t}^{F}\right)$11:  
**end if**
12:**end for**13:**return**$L_{T}$ and $L_{F}$


### 2.5. Fusion of Terrain Predictions

In ensemble learning, voting, including the majority, plurality, and weighted voting, are general schemes to fuse different predictions [45]. However, they cannot be used to our fusion task directly, since we only have two domains. Two dedicated schemes follow:

The 1st fusion scheme is

(9) $$\begin{array}{c} o_{t}^{1}=\left\{\begin{array}{cc} {\hat{c}}_{t}^{T}, & \mathrm{if}\;\;{\hat{p}}_{t}^{T}>w{\hat{p}}_{t}^{F},\\ {\hat{c}}_{t}^{F}, & \mathrm{if}\;\;{\hat{p}}_{t}^{T}\leq w{\hat{p}}_{t}^{F}, \end{array}\right. \end{array}$$

where $o_{t}^{1}$ denotes the fused terrain prediction using the 1st fusion scheme, ${\hat{p}}_{t}^{\gamma }$ denotes the confidence of ${\hat{c}}_{t}^{\gamma }$. The weight w>0 assigns the two *a posteriori* terrain predictions different weights, which are often set as a number larger than 1 because the frequency domain usually outperforms the time domain.

The 2nd fusion scheme is

(10) $$\begin{array}{cc} \; & o_{t}^{2}=M\left\{\frac{{\hat{v}}_{t}^{T}+w{\hat{v}}_{t}^{F}}{1+w}\right\}, \end{array}$$

where $o_{t}^{2}$ denotes the fused terrain prediction using the 2nd fusion scheme, ${\hat{v}}_{t}^{\gamma }$ denotes the confidence vector of the γ-domain Bayesian filtering at time *t*. The weight w>0 should be a number larger than 1. The function M{·} returns the index of the largest element in the vector. As a result that the terrain IDs correspond to the vector indices, M{·} returns the terrain prediction.

The mentioned fusion schemes fuse the *a posteriori* terrain predictions, while they can be also used to fuse the *a priori* terrain predictions.

## 3. Experimental Verification

In this section, we first present the description of the experimental robot, the experimental terrains, and the details of the experimental data collection. Second, we demonstrate the performance of the traditional terrain classification methods when data drift exists. Thirdly, we exhibit how the Bayesian filtering improves the classification results. Finally, a comparative study is done to verify the effectiveness of the proposed DyVTC.

### 3.1. Experimental Data Collection

**Figure 2:**
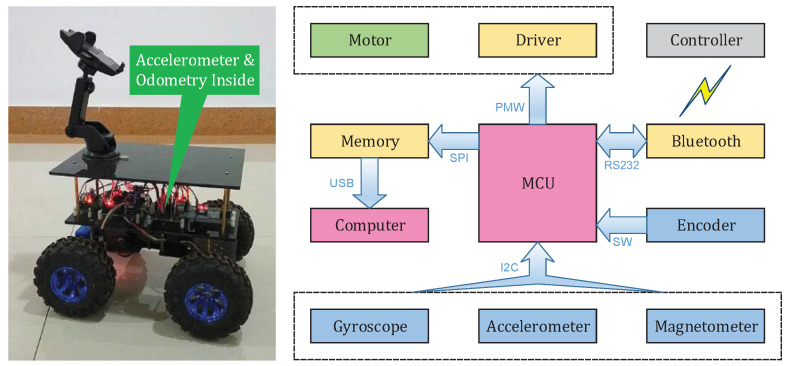
A four-wheeled mobile robot for experiment. The left figure shows the robot photograph, and the right one shows its electronic system structure and signal flows.

The experimental robot and its electronic system structure and signal flows are shown in Figure 2. The robot is 340 mm in length, 270 mm in width, 230 mm in height, and 2.6 kg in mass. The diameter and width of the wheels are 130 mm and 60 mm, respectively. With a power supply of 12 V, the robot could traverse coarse grounds at the speed of up to 1.5 m/s. An accelerometer–gyroscope–magnetometer integrated sensor (MPU9250) and an odometry constitute the sensor system. The main configurations of odometry, gyroscope, accelerometer, and magnetometer are exhibited in Table 2. The odometry is actually four incremental encoders which are directly mounted on the motor shafts to perceive the motor rotational speeds; and consequently, the odometry outputs the robot’s moving speed. The accelerometer–gyroscope–magnetometer integrated sensor can be used to obtain the robot pose and the vibration. The micro control unit (MCU) reads the Z-axis accelerometer at 100 Hz. Meanwhile, the robot moving speed and pose are measured every second, which evaluates the robot motion modes. The robot is controlled with a smart phone, by sending commands to the robot via Bluetooth. The MCU is a development board of Arduino Mini Pro which is used to realize some simple and fundamental operations, such as data gathering, motor control, and command receiving. While the robot is working, all data are stored in the local memory (a T-Flash card); and next, the card is unplugged from the robot, connected, and transferred to a desktop computer (3.20 GHz, 8 GB RAM).

**Figure 3:**
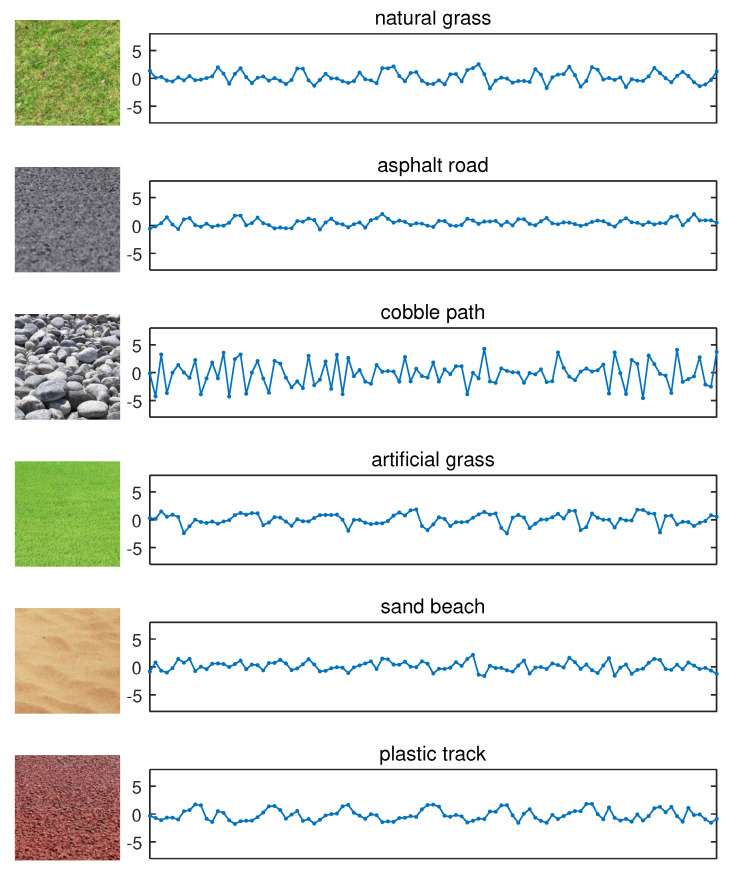
Photos of the traversed terrains and the corresponding segments of vibration time series. From top to bottom, the experimental terrains are: natural grass, asphalt road, cobble path, artificial grass, sand beach, plastic track, respectively. They are abbreviated as NG, AR, CP, AG, SB, and PT, respectively. The *Y* axis represents acceleration (m/s${}^{2}$).

All algorithms will be evaluated on the computer based on the gathered data. Among the terrains listed in [46], we select six terrains on which a robot is most likely to traverse to do the experiment. As shown in Figure 3, some of them are artificial terrains (e.g., asphalt road), while some are natural ones (e.g., natural grass). These terrains are different in rigidity, roughness, and flatness. The segments of vibration time series collected on the six terrains and the corresponding terrain photographs are shown in Figure 3. Compared with other terrains, it is observed that the interaction between the robot and the cobble path generates a highly distinguishable vibration. The vibration has higher frequency, larger magnitude, and weaker autocorrelation, because the cobble path is relatively rigid and irregular. The vibrations of the other five terrains may not be easy to discriminate intuitively because of their slight differences; however, they still can be found in terms of their variation tendency.

**Figure 4:**
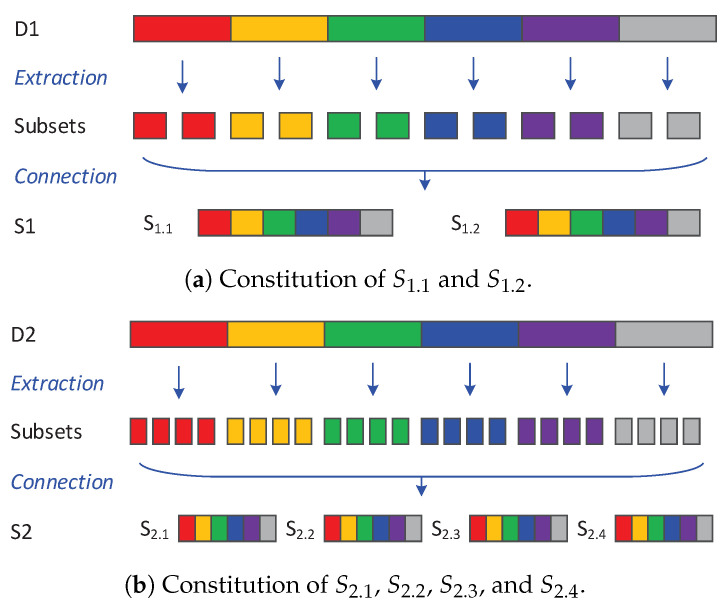
Illustration of data constitution.

Different motion states might also cause the data drift, but it could be eliminated by the sufficient data collection, as the number of motion states are relatively limited. Hence, in our experiment of data collection, we control the experimental robot to wander on the six terrains at a speed ranging from the minimum speed (0.2 m/s) to the maximum speed (1.1 m/s) and in different motion modes (e.g., circular and linear motion), which avoids the data drift from an insufficient experiment. We collect the vibration data in two different environments, thus obtaining two vibration datasets: $D_{1}$ and $D_{2}$. lIntuitively speaking, (i) the grass in garden and roadside may be different in height, (ii) the natural grass gets harder under fine weather, while softer after raining, and (iii) the soil is harder at night than that in the daytime because of the lower temperature. Environment One and Environment Two both include the aforementioned 6 terrains, but are different in location, weather, and temperature. For each dataset, the vibration time series are segmented into vibration frames by every 100 points with 50% overlap, and therefore, $D_{1}$ and $D_{2}$ are transformed into $S_{1}$ and $S_{2}$ which are composed of vibration frames. As shown in Figure 4a, $S_{1}$ is divided into $S_{1.1}$ and $S_{1.2}$, each of which contains 3000 frames. Similarly, as shown in Figure 4b, $S_{2}$ is divided into $S_{2.1}$, $S_{2.2}$, $S_{2.3}$, and $S_{2.4}$, each of which contains 3000 frames. In addition, according to different feature extraction methods, $S_{1.1}$ is transformed into two sample sets, where $S_{1.1}^{T}$ and $S_{1.1}^{F}$ are derived by using time-domain features and frequency-domain features, respectively. Analogously, we have $\left(S_{1.2}^{T},S_{1.2}^{F}\right)$, $\left(S_{2.1}^{T},S_{2.1}^{F}\right)$, $\left(S_{2.2}^{T},S_{2.2}^{F}\right)$, $\left(S_{2.3}^{T},S_{2.3}^{F}\right)$, and $\left(S_{2.4}^{T},S_{2.4}^{F}\right)$.

### 3.2. Performance Evaluation of Classifier

To evaluate the classifier performance in a static environment, i.e., the training and test data are both gathered in Environment One, we train two classifiers on $S_{1.1}^{T}$ and $S_{1.1}^{F}$, and test them on $S_{1.2}^{T}$ and $S_{1.2}^{F}$, respectively. The Gaussian kernel is employed for the time-domain classifier. As for the frequency-domain classifier, because the feature vector is of high dimension, we employ a linear kernel. We use the confusion matrix to show the classification performance. The rows of the confusion matrices represent the real terrains, while the columns represent the predicted terrains. The trained time- and frequency-domain SVM model can achieve the accuracies of 85.4% and 86.5%, which are acceptable to a field robot. It is observed that the main confusion exists between the terrains of natural gas (NG) and sand beach (SB). Compared with other terrain, NG and SB are both natural terrains, and have the similar rigidity and unevenness. In addition, the terrain of plastic track (PT) cannot be easily classified. The classifier $C_{T}$ cannot distinguish PT and asphalt road (AR) perfectly, while $C_{F}$ are confused in PT and artificial grass (AG). The terrains of PT, AR, and AG are all artificial terrains, which are made to enhance pedestrian or vehicle’s traversability, so they usually have the similar characteristics in rigidity, roughness, and flatness.

**Figure 5:**
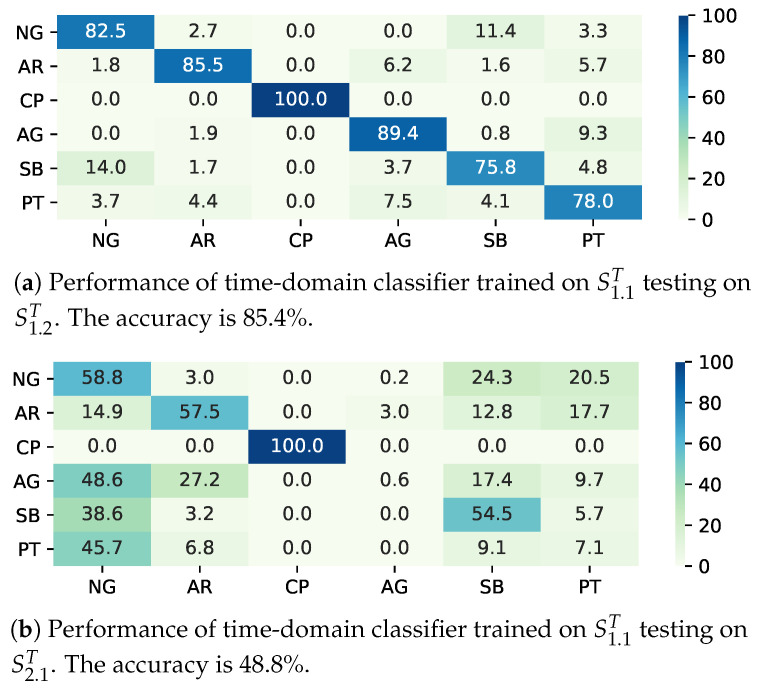
Normalized confusion matrices (in %) of SVM-based terrain classification on sample set $S_{2}$. NG, AR, CP, AG, SB, PT denote natural grass, asphalt road, cobble path, artificial grass, sand beach, plastic track, respectively.

**Figure 6:**
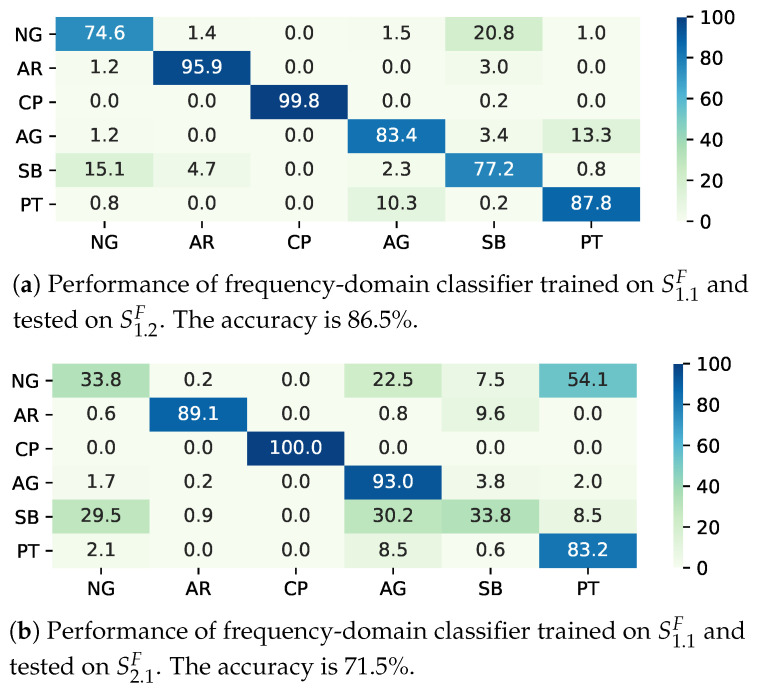
Normalized confusion matrices (in %) of SVM-based terrain classification on a sample set $S_{2}$. NG, AR, CP, AG, SB, PT denote natural grass, asphalt road, cobble path, artificial grass, sand beach, plastic track, respectively.

To evaluate the classifier performance in a dynamic environment, we use the classifiers trained on $S_{1.1}^{T}$ and $S_{1.1}^{F}$ to predict $S_{2.1}^{T}$ and $S_{2.1}^{F}$, respectively. Due to the data drift, the accuracies on $S_{2.1}^{T}$ and $S_{2.1}^{F}$ could only reach 48.8% and 71.5%, respectively. As illustrated in Figure 5a,b, the data drift causes many confusions. In the time domain, only 0.6% of AG samples and 7.1% of PT samples could be classified correctly. Most AG samples are misclassified as NG, AR, and SB. In particular, 45.7% of the PT samples are misclassified as NG. Obviously, the SVM model trained on $S_{1.1}^{T}$ cannot distinguish AG from other terrains on $S_{2}^{T}$. In the frequency domain, as demonstrated in Figure 6a,b, the classifier performs much better under data drift, but only about 33% of NG and SB samples can be classified correctly. The performance degradation of SVM model is caused by data drift. In our experiment, NG is the most changeful terrain, hence becoming the main class that confuses the classifier.

**Figure 7:**
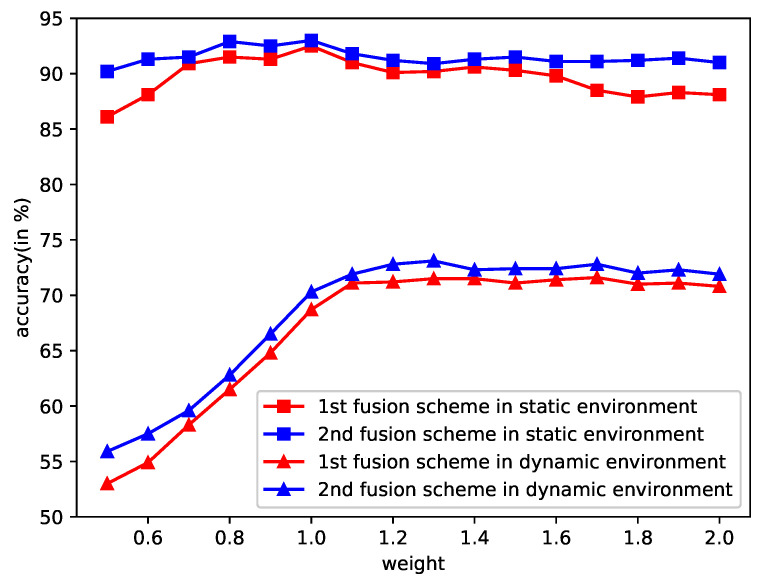
Fusion accuracies of the time- and frequency-domain classifiers with different weights.

The fusion accuracies with different *w* are shown in Figure 7. It is observed that the fusion of the time- and frequency-domain classifiers could increase the classification accuracy slightly, with an appropriate *w*. The time-domain classifier performs much worse than the frequency-domain one, so the increase of fusion accuracy is not significant.

The offline terrain classification, which means the classifiers performing on $S_{1}^{T}$/$S_{1}^{F}$, could achieve a maximum accuracy of 92.7%. The offline classification accuracy is improved. However, the online terrain classification, which means the classifiers performing on $S_{2}^{T}$/$S_{2}^{F}$, does not see a significant improvement. The online classification accuracy can be increased by about only 1% if ω could be appropriately set. If we have no *a priori* knowledge on the two views and do not know which is better, then the coefficient ω is usually assigned by 1.

### 3.3. Performance Evaluation of Bayesian Filter

**Figure 8:**
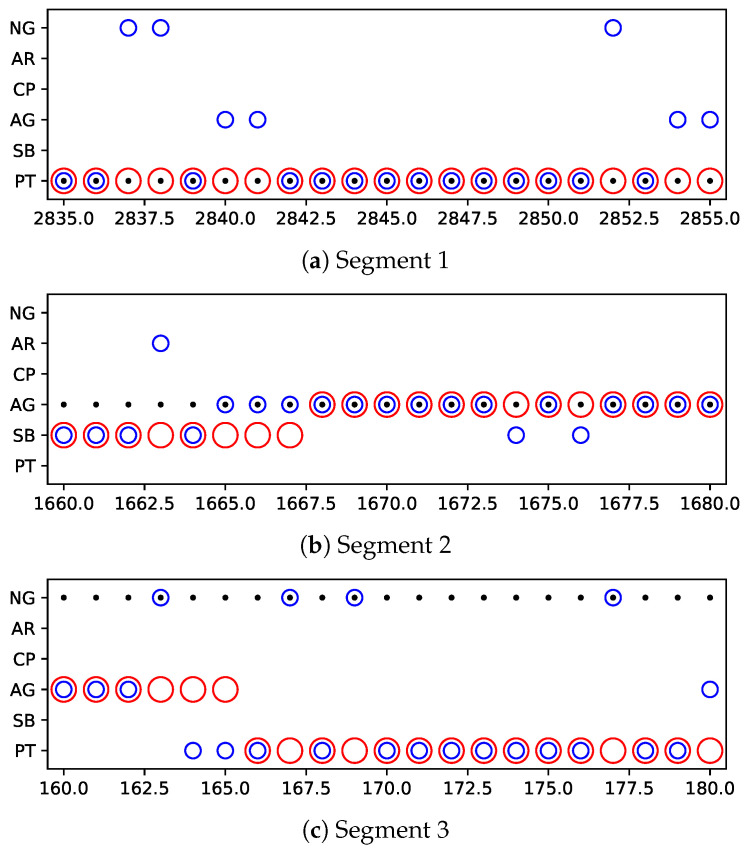
Filtering details exhibition. • denotes real terrain, ∘ denotes classifier-output terrain predictions, ∘ denotes filter-output terrain predictions. NG, AR, CP, AG, SB, PT denote natural grass, asphalt road, cobble path, artificial grass, sand beach, plastic track, respectively.

Now we are in the position to evaluate the Bayesian filter improving the classifier-output terrain predictions. Here, we exhibit the details of the Bayesian filter correcting the classifier’s outputs, as shown in Figure 8. Taking the temporal correlation in sample stream into consideration, the prediction of the current terrain is not only based on the current vibration frame any more, but a combination of the current vibration frame and the previous terrain prediction. Hence, as shown in Figure 8a,b, the incorrect predictions by the classifier at time 1674, 1676, 2837, 2838, 2840, 2841, 2852, 2854, 2855 can be corrected by the Bayesian filter. The Bayesian filter regards the classifier-output terrain predictions as observations. Due to the introduction of temporal correlation, which results in the lags in response to the variation of observations, the Bayesian filter outputs incorrect predictions at time 1663, 1665–1667, as shown in Figure 8b. Such lags can be found in Figure 8c as well. Furthermore, it is known that the Bayesian filter has the ability of tracking the observations. Therefore, as the Figure 8c demonstrated, the Bayesian filter fails if the classifier outputs incorrect terrain predictions continuously.

**Figure 9:**
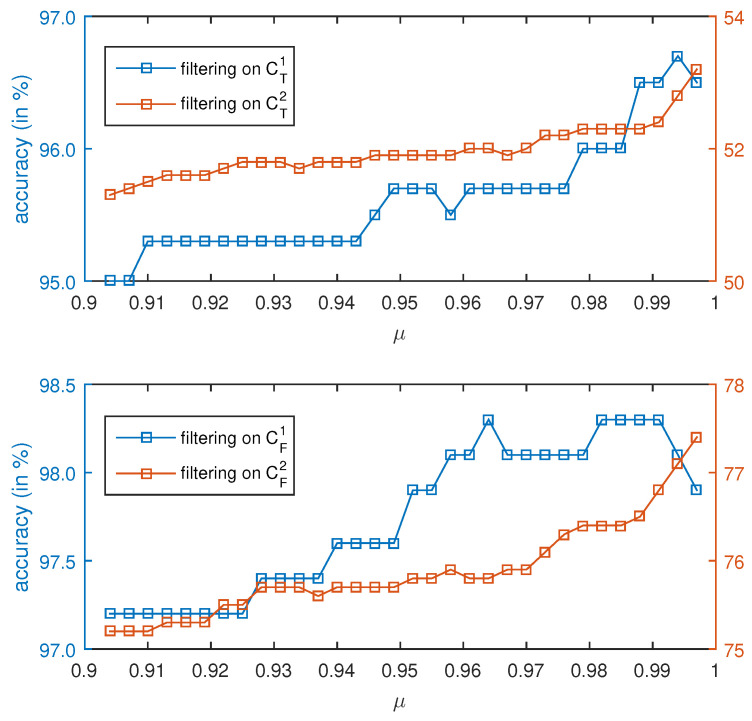
Accuracies of filtering results with different μ. The upper figure is for time domain, while the lower one for frequency domain.

Denote the sets $C_{1}^{T}$, $C_{1}^{F}$, $C_{2}^{T}$, and $C_{2}^{F}$ by the outputs of $C_{T}\left(S_{1}^{T}\right)$, $C_{F}\left(S_{1}^{F}\right)$, $C_{T}\left(S_{2}^{T}\right)$, and $C_{F}\left(S_{2}^{F}\right)$, where $S_{1}^{T}\subset S_{1}^{T}$ and $S_{1}^{F}\subset S_{1}^{F}$ denote the testing set of $S_{1}^{T}$ and $S_{1}^{F}$, respectively. Feeding these classifier’s output set into the Bayesian filter, the classification results are increased by approximately 5% to 10%. The filtering accuracies with different μ are exhibited in Figure 9. It is observed that the accuracies almost reach 97% and 98% with the Bayesian filter performing on $C_{1}^{T}$, $C_{1}^{F}$, which means the offline classification accuracy increases by approximately 10%. On the other hand, filtering on $C_{2}^{T}$ and $C_{2}^{F}$ does not see such an effectiveness, which increases the classification by approximately 7% only.

**Figure 10:**
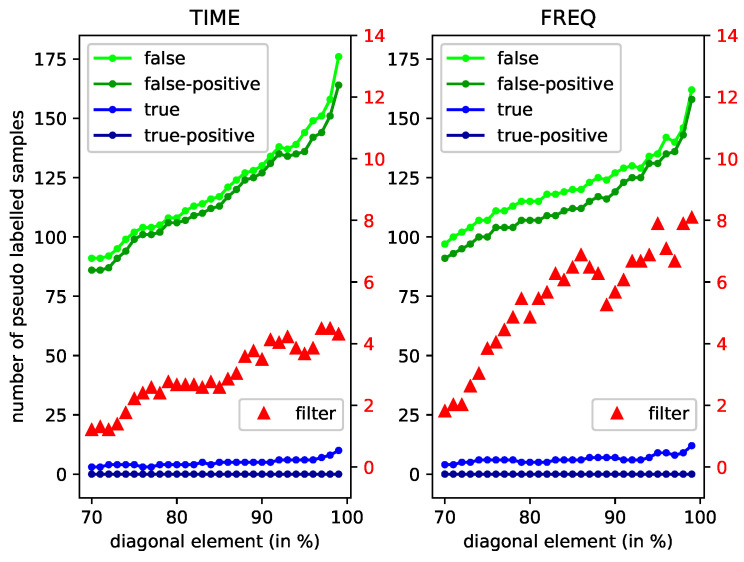
The influences on Bayesian filtering and pseudo-labeling algorithm of different diagonal elements. The right axis corresponds to *filter*. The left axis corresponds to *false*, *false-positive*, *true*, *true-positive*. The left figure is for time domain, while the right one for frequency domain.

The influences on Bayesian filtering and pseudo-labeling algorithm of different diagonal elements are shown in Figure 10. The term “*true*” means the number of the extracted samples that are not key samples (i.e., the samples that can be classified correctly), while “*false*” means the number of key samples (i.e., the samples that cannot be classified correctly). The term “*all*” means the number of the extracted samples. The terms “*true-positive*”, “*false-positive*”, and “*all-positive*” mean the numbers of the *true* samples, *false* samples, and *all* samples which could be correctly labeled by the proposed pseudo-labeling algorithm, respectively. It is observed that the Bayesian filter could increase the classification accuracy to some extent. In the time domain, the increasing accuracy varies from 1.6% to 4%, and peaks when the diagonal element exceeds 97%. Such an accuracy promotion could be found in the frequency domain more apparently. Furthermore, the pseudo-labeling algorithm could extract more *false* and *false-positive* samples with the diagonal element getting larger, both in the time and frequency domain. On the contrary, the number of *true* and *true-positive* samples does not increase significantly. Therefore, the pseudo-labeling algorithm could reach a high performance with a larger diagonal element.

### 3.4. Comparative Study of Adaptation in a Dynamic Environment

As shown above, the classifier trained on $S_{1}$ cannot achieve a high accuracy on $S_{2}$ for the presence of data drift. Now we are in the position to evaluate how DyVTC could retrieve the classification accuracy by incremental learning on the data chunks. As aforementioned, the terrain classification in a dynamic environment has rarely been investigated. We construct some terrain classification methods by applying the existing learning algorithms. The performances of the proposed DyVTC and those constructed ones will be evaluated. The 9 methods are shown as follows:

- *IE1:* The proposed DyVTC. IE is the abbreviation of *in- and ex-disagreement*.
- *IE2:* Similar to IE1, we use the *a priori* ex-disagreement, instead of *a posteriori* ex-disagreement.
- *IE3:* Similar to IE1, we use both *a priori* and *a posteriori* ex-disagreement, which are combined using logical OR.
- *CT.95:* Using co-training algorithm (see [47]) to tackle such a terrain classification problem. The confidence threshold is 0.95.
- *CT.8:* Similar to CT.95, but the confidence threshold is 0.8.
- *ST.95:* Using the self-training algorithm for both domains. The similar idea can be found in [35,36]. The confidence threshold is 0.95.
- *ST.8:* Similar to ST.95, but the confidence threshold is 0.8.
- *KM.95:* Using an advanced fuzzy *k* means (see [48]) semi-supervised clustering algorithm to label the newly collected samples. The confidence threshold is 0.95.
- *KM.8:* Similar to KM.95, but the confidence threshold is 0.8.

**Figure 11:**
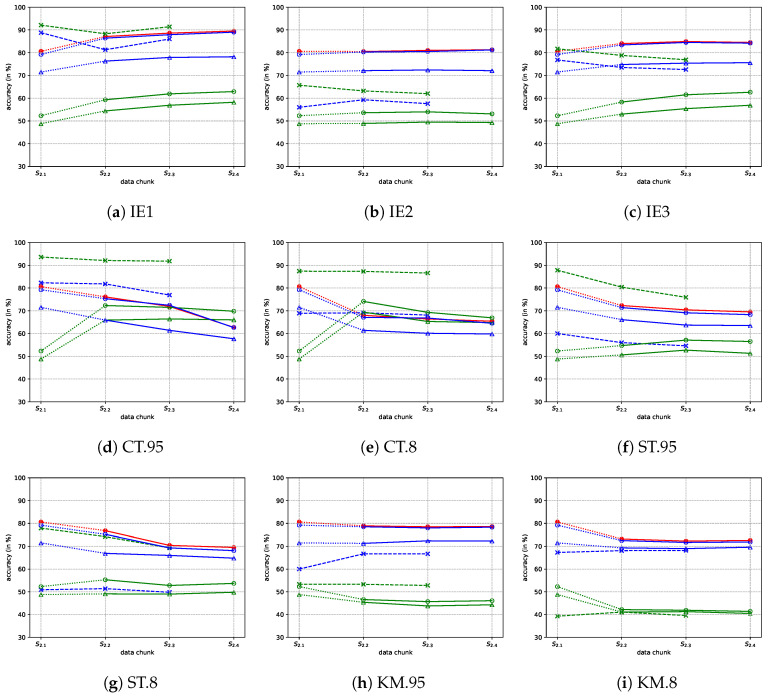
Accuracies of iterative incremental learning. The pseudo-labeling algorithm is conducted on $S_{2.1}$, $S_{2.2}$, and $S_{2.3}$, while the classifier is re-trained incrementally at the end of $S_{2.1}$, $S_{2.2}$, and $S_{2.3}$. The original classifier which is trained on $S_{1.1}$ is tested on $S_{2.1}$, while the updated classifiers are tested on $S_{2.2}$, $S_{2.3}$, and $S_{2.4}$. The fusion is based on the 2nd scheme. The marker definitions follow: • denotes fusion accuracy; ∘, Δ, and * denote the frequency-domain filter, classifier, and pseudo-labeling accuracy, respectively; ∘, Δ, and * denote the time-domain filter, classifier, and pseudo-labeling accuracy, respectively.

The performances of pseudo-labeling algorithms are shown in Table 3. It is observed that the IE1 outperforms all the other algorithms in accuracy. The IE1 algorithm could only extract 100-200 samples from the whole 3000 samples, and the *true-positive* accuracy is 0. However, most of the extracted samples are key samples and these key samples could be labeled in an extremely high accuracy (over 95%). Hence, as shown in Figure 11, such a pseudo-labeling algorithm could increase the classification accuracy on $S_{2}$. The IE2 and IE3 are the variants of IE1. IE2 could extract many *true* samples and label them in 100% accuracy, but its *false-positive* accuracy is 0%. This indicates IE2 cannot bring valuable information, and thus cannot increase nor decrease the classification accuracy. All indices of IE3 are the sums of the corresponding indices of IE1 and IE2, and consequently, the performance of IE3 is between those of IE1 and IE2. We can also observe that the pseudo-labeling accuracies of IE2 and IE3 decrease at learning steps 2 and 3, but the classifier accuracy does not decrease. This is because IE2 and IE3 have high true-positive accuracy, which guarantee that the classifier accuracy does not decrease after update. In conclusion, it is the best to use *a posteriori* ex-disagreement in the pseudo-labeling algorithm.

The CT.95 and CT.8 could increase the accuracy of the time-domain classifier but decrease that of the frequency-domain classifier, which is caused by the unequal performances of the two domains. The frequency-domain classifier performs much better, so it acts as a supervisor of the time-domain classifier. The ST.95 and ST.8 do not utilize a mutual learning mechanism, thus they have no effect on the classifier accuracy. The KM.95 and KM.8 only work under clustering assumption which is seldom satisfied when data drift occurs. Hence, the classifier accuracy decreases after updating using the KM methods. In conclusion, the IE methods could increase the classifier accuracy by incremental learning, but the others cannot work or even are counterproductive.

The time cost is shown in Table 4. It can be observed that IE1, IE2, and IE3 take the shortest time to generate the pseudo-labeled sample set, while KM.95 and KM.8 is the most time-consuming. Unlike KM.95 and KM.8, which could only work after a data chunk is collected completely, IE1, IE2, IE3, CT.95, CT.8, ST.95, ST.8 could generate the pseudo-labeled samples at the time when a vibration frame prediction is finished, so the time cost of pseudo-labeling of these methods could be ignored. For the incremental learning part, compared with CT, ST, and KM, IE1 and IE3 use less pseudo-labeled samples to train the last classifier incrementally, but are the most time-consuming. This is because the majority of the pseudo-labeled samples generated by IE1 and IE3 are correctly-labeled key samples, which leads to the changing of classifier. Even so, IE1 and IE3 could be done within one second, which guarantees the real-time application.

## 4. Conclusions

In this paper, we propose a novel vibration-based terrain classification method for autonomous robots working in a dynamic environment, mainly to suppress the affect rendered by data drift, during the period that manual labels do not arrive. We mainly propose an ex- and in-disagreement-based learning algorithm, which is verified to be powerful to extract key samples and label them in high accuracy. In order to activate such a learning framework, we divide the vibration view into two domains, which may produce ex-disagreements; and introduce the Bayesian filter to correct the classification results, which may produce in-disagreements. The real-world experiment shows that the proposed DyVTC could reach an accuracy of 89.5%, which outperforms the existing VTC methods.

## Figures and Tables

**Table 1:** Entries of the time-domain feature.

Name	Equation	Description
Zero-crossing number (ZCN)	$\phi_{1}=\sum_{i=2}^{n}I\left(a_{i}a_{i-1}<0\right)$	I(·) is an indicator function, which outputs 1 if the expression in (·) holds, or 0 otherwise. This feature is an approximation of the frequency of *a*.
Mean	$\phi_{2}=\frac{1}{n}\sum_{i=1}^{n}a_{i}$	Although the gravitational acceleration has been subtracted, the mean of *a* may considerably diverge from zero for some course terrains.
ZCN in $\bar{a}$	$\phi_{3}=\sum_{i=2}^{n}I\left({\bar{a}}_{i}{\bar{a}}_{i-1}<0\right)$	${\bar{a}}_{i}=a_{i}-\phi_{2}$. $\phi_{3}$ is a complement to $\phi_{1}$, which avoids $\phi_{1}\approx 0$ for even high-frequency vibration signal when the robot is traversing coarse terrains.
Variance	$\phi_{4}=\frac{1}{n}\sum_{i=1}^{n}{\left(a_{i}-\phi_{2}\right)}^{2}$	Intuitively, the variance is higher when the terrain becomes coarser.
Autocorrelation	$\phi_{5}=\frac{1}{\left(n-\tau \right)\phi_{4}}\sum_{i=1}^{n-\tau }\left(a_{i}-\phi_{2}\right)\left(a_{i+\tau }-\phi_{2}\right)$	τ<n is an integer indicating time difference. As a measure of non-randomness, $\phi_{5}$ gets larger with a stronger dependency between $a_{i}$ and $a_{i+\tau }$.
Maximum	$\phi_{6}=\max\left(a\right)$	$\phi_{6}$ indicates the biggest bump of the terrain.
Minimum	$\phi_{7}=\min\left(a\right)$	$\phi_{7}$ indicates the deepest puddle of the terrain.
$\ell_{2}$-norm	$\phi_{8}=\sqrt{\sum_{i=1}^{n}{\left(a_{i}\right)}^{2}}$	$\phi_{8}$ reflects the energy of *a*. If $\phi_{2}\to 0$, $\phi_{9}$ has the similar function as $\phi_{4}$. Instead, we can also use the $\ell_{1}$-norm, i.e., $\phi_{8}^{*}=\sqrt{\sum_{i=1}^{n}\left|a_{i}\right|}$.
Impulse factor	$\phi_{9}=n(\phi_{6}-\phi_{7})/\phi_{8}^{*}$	$\phi_{9}$ measures the impact degree in *a*.
Kurtosis	$\phi_{10}=\frac{1}{n}\sum_{i=1}^{n}{\left(a_{i}-\phi_{2}\right)}^{4}/\phi_{4}^{2}-3$	$\phi_{10}$ measures the deviation degree of the *a* with Gaussian distribution.

**Table 2:** Specifications of sensors.

Sensor	Specifications
Odometry	540 pulse per round; resolution: 0.67 deg.
Gyroscope	range: ±250 deg/s; initial ZRO * tolerance: ±5 deg/s; total RMS ${}^{†}$ noise: 0.1 deg/s.
Accelerometer	range: ±2 g; initial ZGO ${}^{‡}$ tolerance: ±80 mg; total RMS noise: 8 mg.
Magnetometer	range: ±4800 uT.

* zero-rate output; ${}^{†}$ root mean square; ${}^{‡}$ zero-gravity output.

**Table 3:** Comparison of pseudo-labeling algorithms performing on $S_{2.1}$.

		Method								
		IE1	IE2	IE3	CT.95	CT.8	ST.95	ST.8	KM.95	KM.8
**Time** **Domain**	**True** **True-Positive** **Accuracy**	8 0 0%	71 71 **100%**	79 71 89.8%	753 702 93.2%	1005 886 88.2%	716 653 91.2%	1096 868 79.2%	658 610 92.7%	1542 1071 69.5%
	**False** **False-Positive** **Accuracy**	157 152 **96.8%**	37 0 0%	194 152 78.4%	491 462 94.1%	963 834 86.6%	155 112 72.3%	503 378 75.1%	566 43 7.6%	1653 186 11.3%
	**All** **All-Positive** **Accuracy**	165 152 92.1%	108 71 65.7%	273 223 81.7%	1244 1164 **93.6%**	1968 1720 87.4%	871 765 87.8%	1599 1246 77.9%	1224 653 53.3%	3195 1257 39.3%
**Freq** **Domain**	**True** **True-Positive** **Accuracy**	9 0 0%	65 65 **100%**	74 65 87.8%	765 645 84.3%	1232 881 71.5%	1175 708 60.3%	1691 862 51.0%	5 3 60.0%	2298 1985 86.4%
	**False** **False-Positive** **Accuracy**	146 143 **97.9%**	51 0 0%	197 143 72.6%	98 65 66.3%	354 211 59.6%	75 43 57.3%	260 132 50.8%	0 0 0.0%	910 175 19.2%
	**All** **All-Positive** **Accuracy**	155 143 **92.3%**	116 65 56.0%	271 208 76.8%	863 710 82.3%	1586 1092 68.9%	1250 751 60.0%	1951 994 50.9%	5 3 60.0%	3208 2160 67.3%

**Table 4:** Time cost (in microsecond).

Method	Pseudo Labeling	Incremental Learning
IE1	12	584
IE2	13	152
IE3	16	651
CT.95	62	195
CT.8	147	237
ST.95	73	213
ST.8	196	281
KM.95	359	204
KM.8	838	323
